# Serial changes of I-123 FP-CIT SPECT binding asymmetry in Parkinson's disease: Analysis of the PPMI data

**DOI:** 10.3389/fneur.2022.976101

**Published:** 2022-09-01

**Authors:** Eun Hye Jeong, Mun Kyung Sunwoo, Jae Yong Lee, Sun-Ku Han, Sung Wook Hyung, Yoo Sung Song

**Affiliations:** ^1^Department of Neurology, Bundang Jesaeng General Hospital, Seongnam-si, South Korea; ^2^Department of Nuclear Medicine, Seoul National University Bundang Hospital, Seongnam-si, South Korea; ^3^Department of Nuclear Medicine, Seoul National University College of Medicine, Seoul, South Korea

**Keywords:** asymmetry, I-123 FP-CIT, Parkinson's disease, Parkinson's Progression Markers Initiative (PPMI), striatal binding ratio

## Abstract

**Background:**

Dopaminergic denervation and motor symptoms are usually asymmetric at the onset of Parkinson's disease (PD). In this study, we estimated the asymmetry of specific binding ratio (SBR) of I-123 FP-CIT SPECT images during 4-years of follow up, to demonstrate the pattern of serial changes of asymmetry.

**Methods:**

Clinical and I-123 FP-CIT SPECT image data of 301 PD patients and 141 normal controls were reviewed from the Parkinson's Progression Markers Initiative cohort. I-123 FP-CIT SPECT images were taken at baseline, 1-, 2-, and 4-year follow up periods for PD patients, and at baseline for normal controls. Asymmetry index were calculated by two methods. Method 1, by using the ratio of absolute difference of right and left SBRs to the average SBR. Method 2, by using the ratio of absolute difference of right and left SBRs to the SBR values of age-matched normal controls.

**Results:**

Asymmetry index by method 2 revealed a more significant decrease during the 4-year follow up period, compared with method 1. The baseline asymmetry index of the putamen by method 2 showed significant correlation with the non-dominant putamen SBRs. However, there were no significant correlation with the baseline asymmetry index by method 2 and motor symptoms, cognition, nor autonomic symptoms.

**Conclusion:**

We suggest a novel asymmetry index in association to age-matched normal SBR values. This novel index could be adopted in predicting and evaluating the natural course of PD.

## Introduction

Parkinson's disease (PD) is a neurodegenerative disorder known to be caused primarily by the dopaminergic denervation of the nigrostriatal pathway (1). Symptoms, such as resting tremor, bradykinesia, rigidity, and loss of automatic symptoms, occur heterogeneously within patients, and the rate disease progression varies by patient. One of the key characteristics is the asymmetry of dopaminergic denervation at the onset of the disease, presenting a unilateral appearance of motor symptoms (2–5). This asymmetric feature may last for several years, from disease onset until the development of bilateral motor symptoms; however, the degree of symptoms between the bilateral sides may not be balanced throughout the disease progression. To date, there have been several suggestions for the pathological cause of this asymmetry, including inborn variations of dopaminergic innervation in each hemisphere, different vulnerability of the two hemispheres, hand dominance, and the asymmetrical weakening of the blood–brain barrier (3). However, the underlying mechanism remains unclear.

The hemispheric asymmetry is suggested to determine the prognosis by affecting motor performance, but also autonomic dysfunction and cognition (3, 6). Although it has been suggested that the conversion to symmetrical patterns may be associated with a worse prognosis, the degree and persistency of asymmetry are known to vary among PD patients (5). While in some patients there tends to be no significant changes to the asymmetry of symptoms, others show symmetrical distribution of symptoms within a few years of disease onset. Several dopaminergic imaging studies have evaluated the value of asymmetry in improving PD diagnosis (7–9); however, there are no significant studies that have evaluated the pattern of serial changes of asymmetry during PD progression.

In this study, we investigated the asymmetrical patterns of I-123 FP-CIT SPECT binding in the Parkinson's Progression Markers Initiative (PPMI) database. Since the PPMI is a longitudinal, observational study, it provides an analytic dataset of the PD cohort. Herein, we evaluated the serial changes and associated factors of I-123 FP-CIT SPECT binding asymmetry with a 4-year follow up period.

## Materials and methods

### Study participants

Information regarding PD patients and normal controls (NC) from the PPMI database (http://www.ppmi-info.org) were downloaded in February, 2022. The PPMI is a longitudinal, multi-center trial assessing the clinical features, dopamine transporter (DAT) imaging data, and biologic markers of PD patients. The inclusion criteria for both PD patients and NC are described in http://www.ppmi-info.org. For PD patients, patients with concordant symptoms, diagnosis of PD for 2-years or less at screening, Hoehn and Yahr (H&Y) stage I or II at baseline, confirmation of dopamine image abnormalities, no PD medication within at least 6 months from baseline, and patients—male or female—aged 30 years or older at the time of diagnosis were enrolled. For NC, subjects—male or female—aged 30 years or older at the time of screening without any history of drugs that might interfere with dopamine transporter SPECT imaging were enrolled. Therefore, in this analysis, a total of 304 PD patients (age 61.0 ± 9.6, M: F = 199: 105) and 141 NC subjects (age 60.7 ± 11.1, M: F = 88: 53) were enrolled. Other clinical data such as gender, age, disease duration from first related symptom, results of I-123 FP-CIT SPECT imaging, clinical symptoms, levodopa equivalent daily dose (LEDD), and other clinical characteristics were also extrapolated from the PPMI database. Patients were also subtyped according to their dominant motor symptoms, as tremor dominant (TD), indeterminate, and postural instability gait disorder (PIGD) subtypes. Ratio of the mean tremor scores to the mean PIGD scores from the MDS-UPDRS scores at baseline was used for subtyping, with the following criteria; TD patients (ratio ≧ 1.15), PIGD patients (ratio ≦ 0.90), and indeterminate patients (ratios > 0.90 and <1.15) (10). The clinical data were reevaluated at the 1-, 2-, and 4-year follow-up periods. A total of 264 patients were evaluated at the 1- and 2-year follow-ups, and 239 patients were evaluated at the 4-year follow-up mark. The PPMI is performed in accordance with relevant regulations; written informed consent agreement was obtained from all participants. Study was approved by the institutional review boards of 49 clinical sites (https://www.ppmi-info.org/about-ppmi/ppmi-clinical-sites). This study was performed in accordance with the ethical standards of the institutional and/or national research committee and with the 1964 Helsinki declaration and its later amendments or comparable ethical standards.

### I-123 FP-CIT SPECT scans

I-123 FP-CIT SPECT images from respective institutions were managed by the core imaging lab of PPMI. Each institutions received technical setup visits, and the core lab of PPMI performed quality control in order to maintain reliable data quality. I-123 FP-CIT SPECT images were taken 4 ± 0.5 h after I-123 FP-CIT injection (111–185 MBq). Images were reconstructed iteratively, with no filtering. PMOD software (PMOD Technologies, Zurich, Switzerland) was used for the calculation of specific binding ratios [SBRs, (target region/reference region)-1] of bilateral caudate and putamen with the occipital cortex as the reference. In the NC group, average SBR values of the right and left caudate/putamen were used for analysis. In the PD group, SBRs of the dominant hemisphere was used for analysis. The dominant hemisphere was defined as the lateral side with more severe motor symptoms. Asymmetry index was calculated using two methods. The 1st method (method 1) was calculated as: |SBRleft– SBRright|/[(SBRleft + SBRright)/2] ×100. Asymmetry index by method 1 was calculated based on previous studies (7, 11–16). In this study, we adopted a new method for the calculation of the asymmetry index (method 2): |SBRleft– SBRright|/SBR values of age-matched normal controls ×100.

### Statistical analysis

A dedicated software (MedCalc Software, version 20.014, Belgium) was used for statistical analysis. Parametric factors were compared by Student *t*-test or one-way ANOVA with Scheffé test for *post-hoc*, and non-parametric factors with Mann-Whitney test or Kruskal-Wallis test with Conover test for *post-hoc*. Linear regression analysis was done to correlate quantitative values and acquire Pearson's correlation coefficients (*r*). *P*-values of < 0.05 were considered to indicate statistical significance.

## Results

### Study participants' characteristics

**Figure 1:**
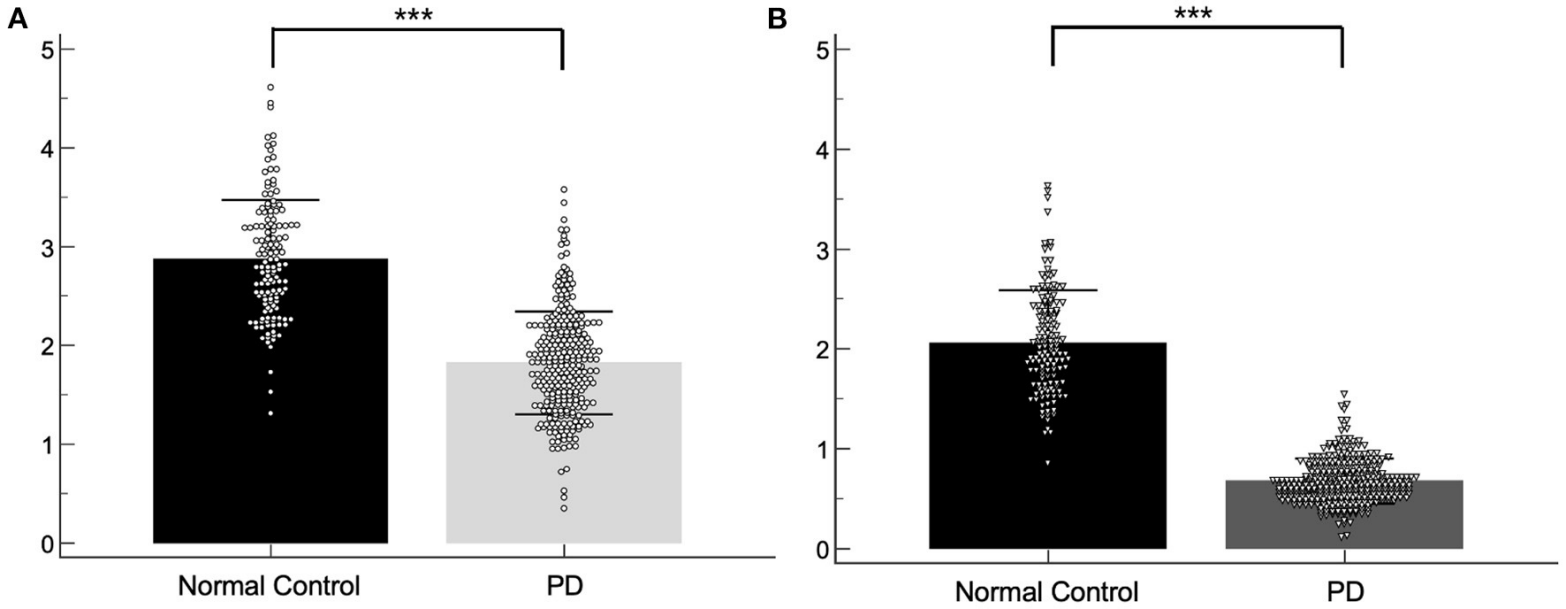
Scatter plots of the baseline SBRs for the normal control and PD group. SBRs of the caudate **(A)** and putamen **(B)**. ***p < 0.001.

Baseline demographic features of the NC group (*n* = 141) and the PD group (*n* = 304) were compared. While there were no significant differences in age, gender, and weight between the two groups, the SBRs of the caudate (2.87 ± 0.60 vs. 1.82 ± 0.52, *p* < 0.001) and putamen (2.01 ± 0.54 vs. 0.67 ± 0.22, *p* < 0.001) were significantly lower in the PD group than in the NC group (Figure 1). The average H&Y scores, MDS-UPDRS II scores, MDS-UPDRS III scores, SCOPA-AUT, and MoCA scores in the PD group were 1.55 ± 0.51, 6.6 ± 4.1, 20.4 ± 8.6, 8.8 ± 5.6, and 27.2 ± 2.3, respectively, which were significantly aggravated than the NC group. Baseline demographic features are presented in Table 1. There were significant differences of SBR values of the NC group according to gender for both caudate (M: F = 2.78 ± 0.54: 3.01± 0.67, *p* < 0.05) and putamen (M: F = 1.98 ± 0.50: 2.17 ± 0.58, *p* < 0.05). There were no significant differences of baseline SBR values of the PD group according to gender for both caudate (M: F = 1.82 ± 0.53: 1.82 ± 0.51) and putamen (M: F = 0.68 ± 0.22: 0.66 ± 0.22). For the PD group, SBRs of the caudate and putamen, average H&Y scores, MDS-UPDRS II scores, MDS-UPDRS III scores, and SCOPA-AUT tended to aggravate during the 4-year follow-up period. The demographic features of the PD group during each follow-up periods are presented in Table 2.

**Table 1:** Characteristic features of the NC group and the PD group.

	**Normal control** **(*n* = 141)**	**PD** **(*n* = 304)**	***p-*value**
Age at enrollment (years)	60.7 ± 11.1	61.0 ± 9.6	0.77
Disease duration (years)	-	2.0 ± 1.8	
Gender (Male: Female)	88: 53	199: 105	0.58
Weight (kg)	78.5 ± 15.8	81.3 ± 17.1	0.10
Caudate nucleus SBRs	2.87 ± 0.60	1.82 ± 0.52	<0.001
Putamen SBRs	2.01 ± 0.54	0.67 ± 0.22	<0.001
H&Y scores	0.01 ± 0.01	1.55 ± 0.51	<0.001
MDS-UPDRS II scores	1.5 ± 1.1	6.6 ± 4.1	<0.001
MDS-UPDRS III scores	1.24 ± 2.1	20.4 ± 8.6	<0.001
SCOPA-AUT	5.4 ± 3.5	8.8 ± 5.6	<0.001
MoCA scores	28.3 ± 1.1	27.2 ± 2.3	<0.001

**Table 2:** Clinical data of the PD group at each follow-up timepoints.

	**Baseline**	**1-year**	**2-year**	**4-year**	***p-*value**
Caudate nucleus SBRs	1.82 ± 0.52^a)^	1.67 ± 0.48^b)^	1.54 ± 0.50^c)^	1.37 ± 0.49^d)^	<0.001
Putamen SBRs	0.67 ± 0.22^a)^	0.61 ± 0.22^b)^	0.57 ± 0.21^b)^	0.50 ± 0.19^c)^	<0.001
H&Y scores	1.55 ± 0.51^a)^	1.69 ± 0.53^b)^	1.77 ± 0.56^b)^	1.96 ± 0.53^c)^	<0.001
MDS-UPDRS II scores	6.6 ± 4.1^a)^	8.0 ± 4.5^b)^	8.7 ± 5.0^c)^	10.6 ± 6.5^d)^	<0.001
MDS-UPDRS III scores	20.4 ± 8.6^a)^	23.1 ± 10.1^b)^	25.2 ± 11.2^c)^	30.2 ± 11.9^d)^	<0.001
SCOPA-AUT	8.8 ± 5.6^a)^	9.7 ± 6.7^b)^	12.3 ± 6.9^c)^	12.4 ± 7.1^c)^	<0.001
MoCA scores	27.2 ± 2.3^a)^	26.6 ± 2.7^b)^	26.5 ± 2.7^b)^	26.7 ± 3.2	<0.05

For a particular variable, values with different superscripts indicate statistically significant difference.

### Asymmetry index during the follow up period

**Figure 2:**
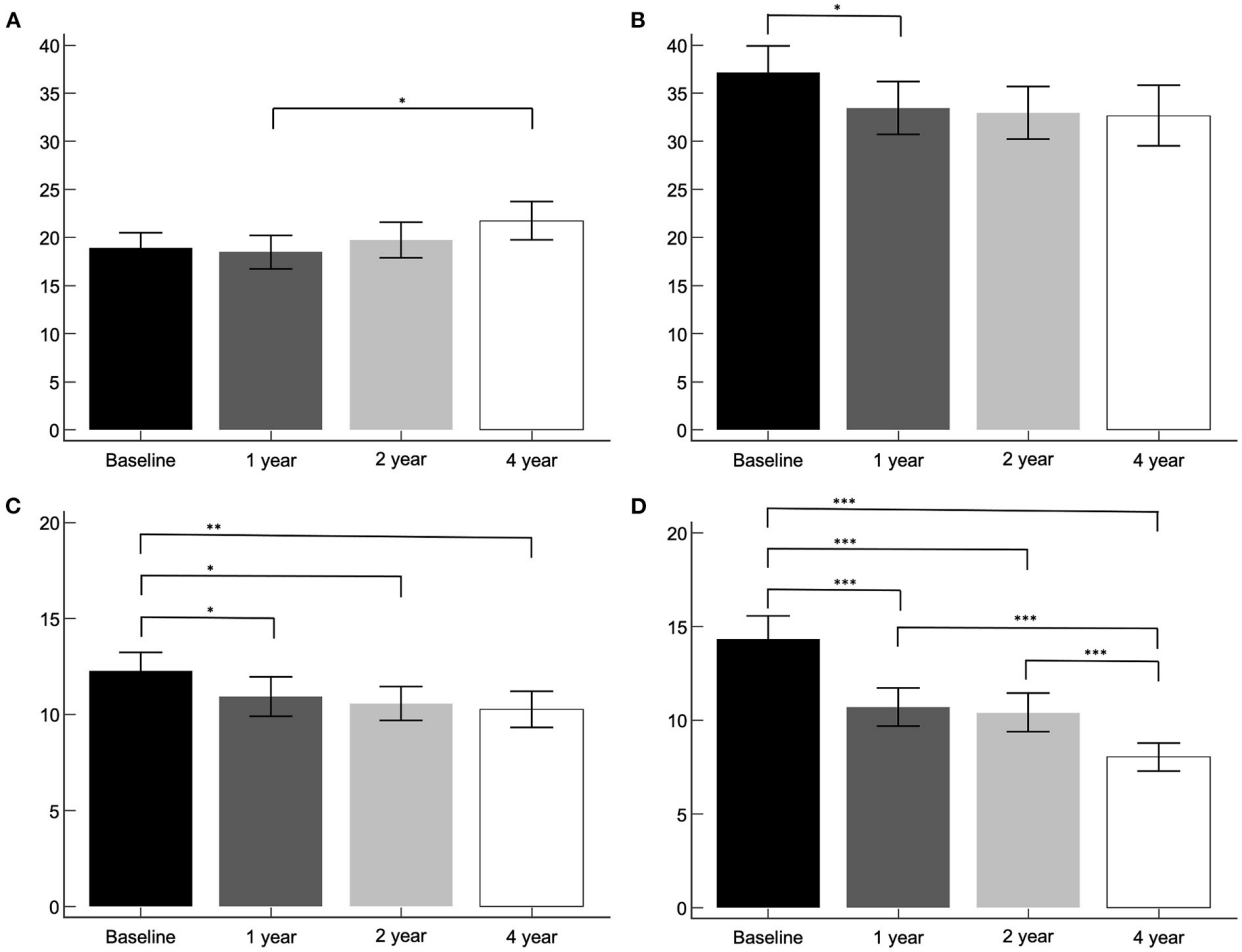
The asymmetry index of the PD group. Asymmetry index by method 1, caudate **(A)** and putamen **(B)**. Asymmetry index by method 2, caudate **(C)** and putamen **(D)**. *p < 0.05, **p < 0.01, ***p < 0.001.

The asymmetry index of the PD group was compared between the baseline, 1-, 2-, and 4-year I-123 FP-CIT SPECT images (Figure 2). Asymmetry index by method 1 revealed a significant increase in the caudate between the 1- and 4-year images (*p* < 0.05), and a significant decrease in the putamen between the baseline and 1-year images (*p* < 0.05). However, asymmetry index by method 2 revealed a significant decrease in the caudate between the baseline and 1- (*p* < 0.05), 2- (*p* < 0.05), and 4-year (*p* < 0.05) images. The putamen showed a significant decrease throughout the follow-up period (*p* < 0.001), except between the 1- and 2-year images.

### Asymmetry index by method 2 according to different clinical subtypes and gender

**Figure 3:**
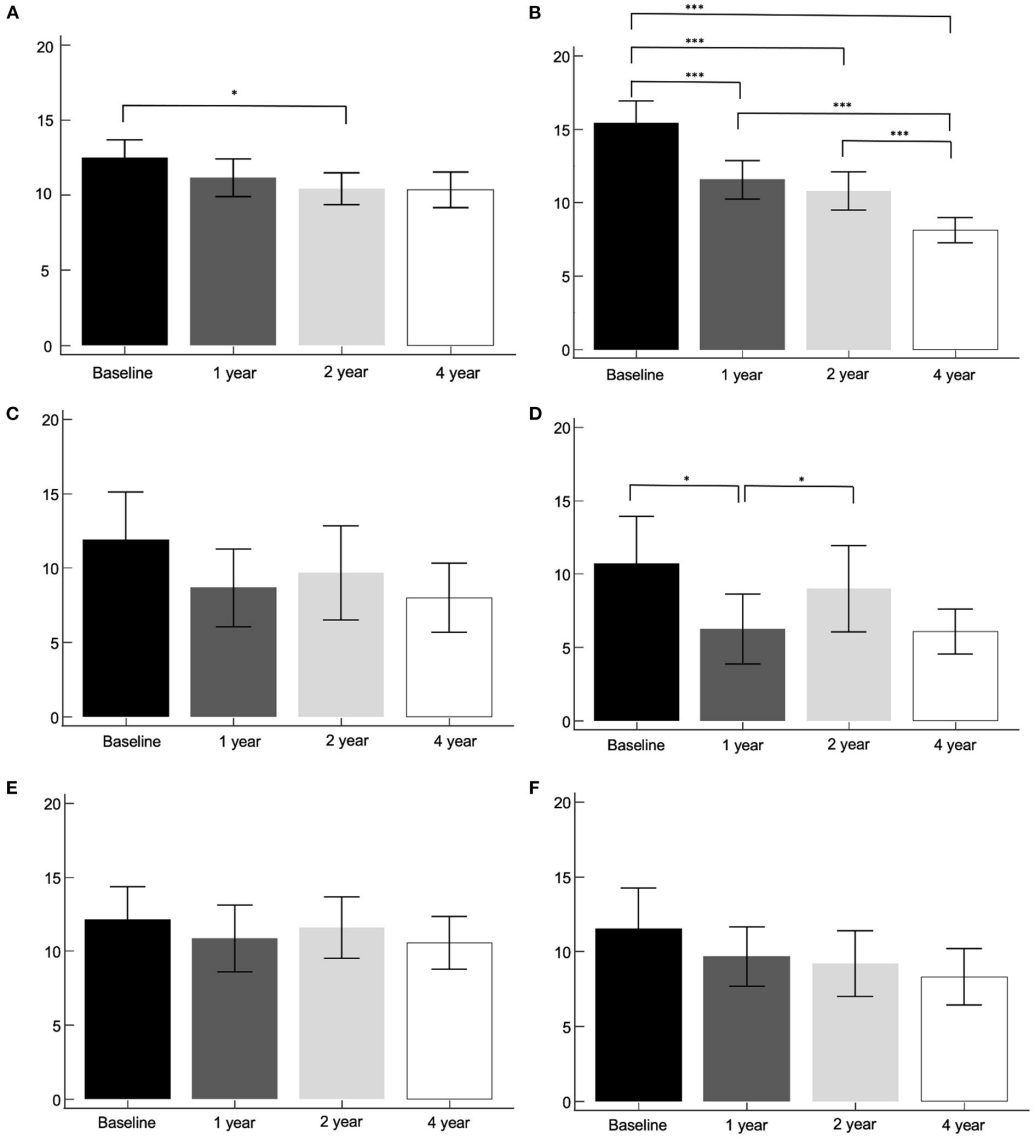
The asymmetry index by method 2, according to clinical subtypes. Tremor dominant subtype, caudate **(A)** and putamen **(B)**. Indeterminant subtype, caudate **(C)** and putamen **(D)**. PIGD subtype, caudate **(E)** and putamen **(F)**. *p < 0.05, ***p < 0.001.

Two hundred and eleven patients were subtyped as the TD group, 27 patients as the indeterminant group, and 66 patients as the PIGD group. The asymmetry index by method 2, according to clinical subtypes were compared between the baseline, 1-, 2-, and 4-year I-123 FP-CIT SPECT images (Figure 3). The tremor dominant subtype revealed a significant decrease in the caudate between the baseline and 2-year images. The putamen showed a significant decrease throughout the follow-up period (*p* < 0.001), except between the 1- and 2-year images. The indeterminant subtype and PIGD subtype did not show significant changes throughout the 4-year follow up. UPDRS III scores, SCOPA scores, and MoCA scores were correlated with the baseline asymmetry index by method 2 in the TD group, the indeterminant group, and the PIGD group. Results were not significantly different with the whole PD group.

There were no significant differences of baseline asymmetry indices by method 1 according to gender for both caudate (M: F = 18.2 ± 13.3: 20.3 ± 13.3) and putamen (M: F = 36.8 ± 24.2: 37.7 ± 25.6), nor of baseline asymmetry indices by method 2 according to gender for both caudate (M: F = 11.9 ± 8.6: 13.0 ± 8.1) and putamen (M: F = 14.4 ± 11.1: 14.2 ± 10.6).

### Correlation of baseline asymmetry index and SBRs

**Figure 4:**
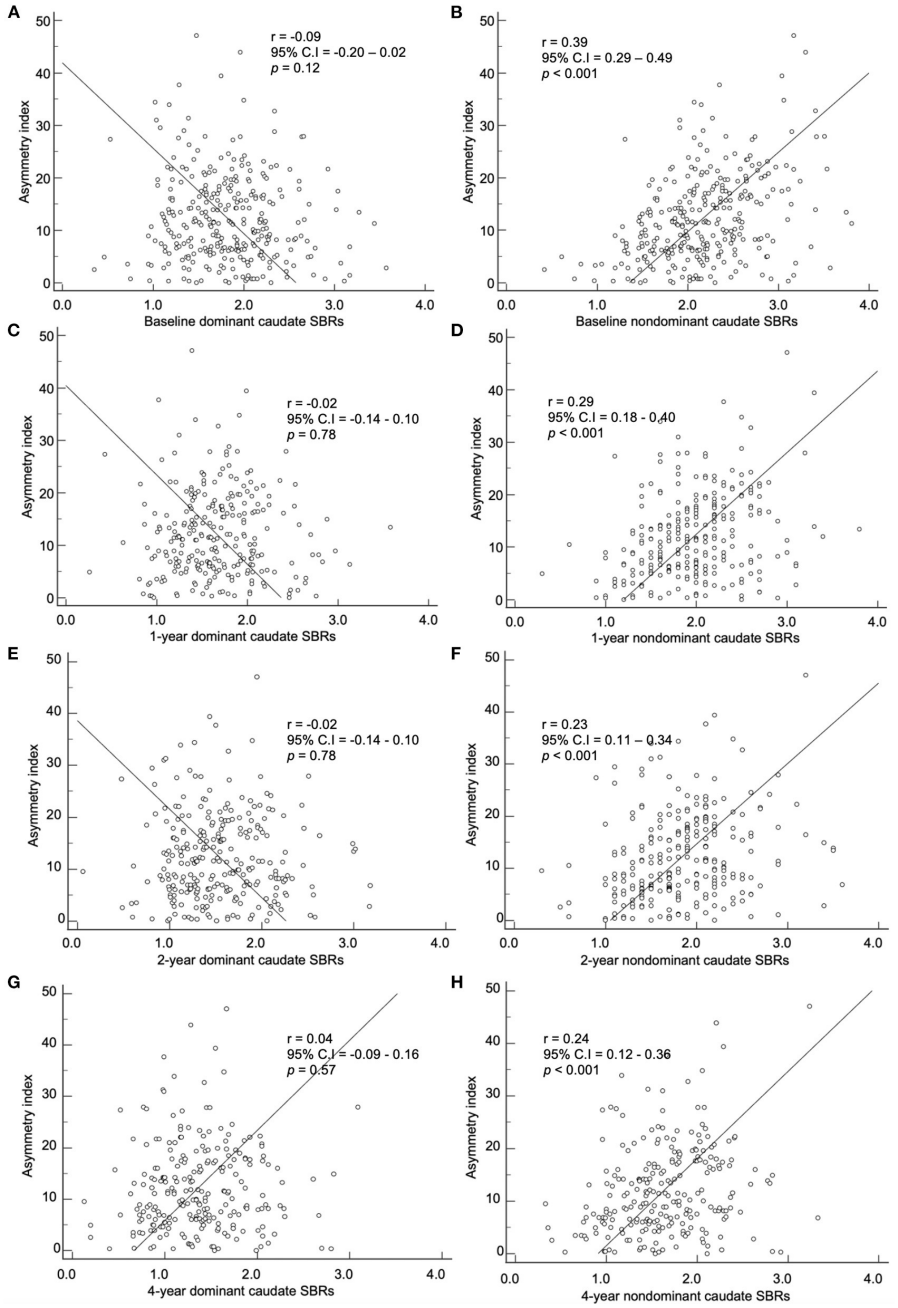
Correlation of the baseline asymmetry index of the caudate by method 2 with SBRs of **(A)** baseline, **(C)** 1-year, **(E)** 2-year, and **(G)** 4-year. Correlation of the baseline asymmetry index of the caudate by method 2 with SBRs of **(B)** baseline, **(D)** 1-year, **(F)** 2-year, and **(H)** 4-year.

**Figure 5:**
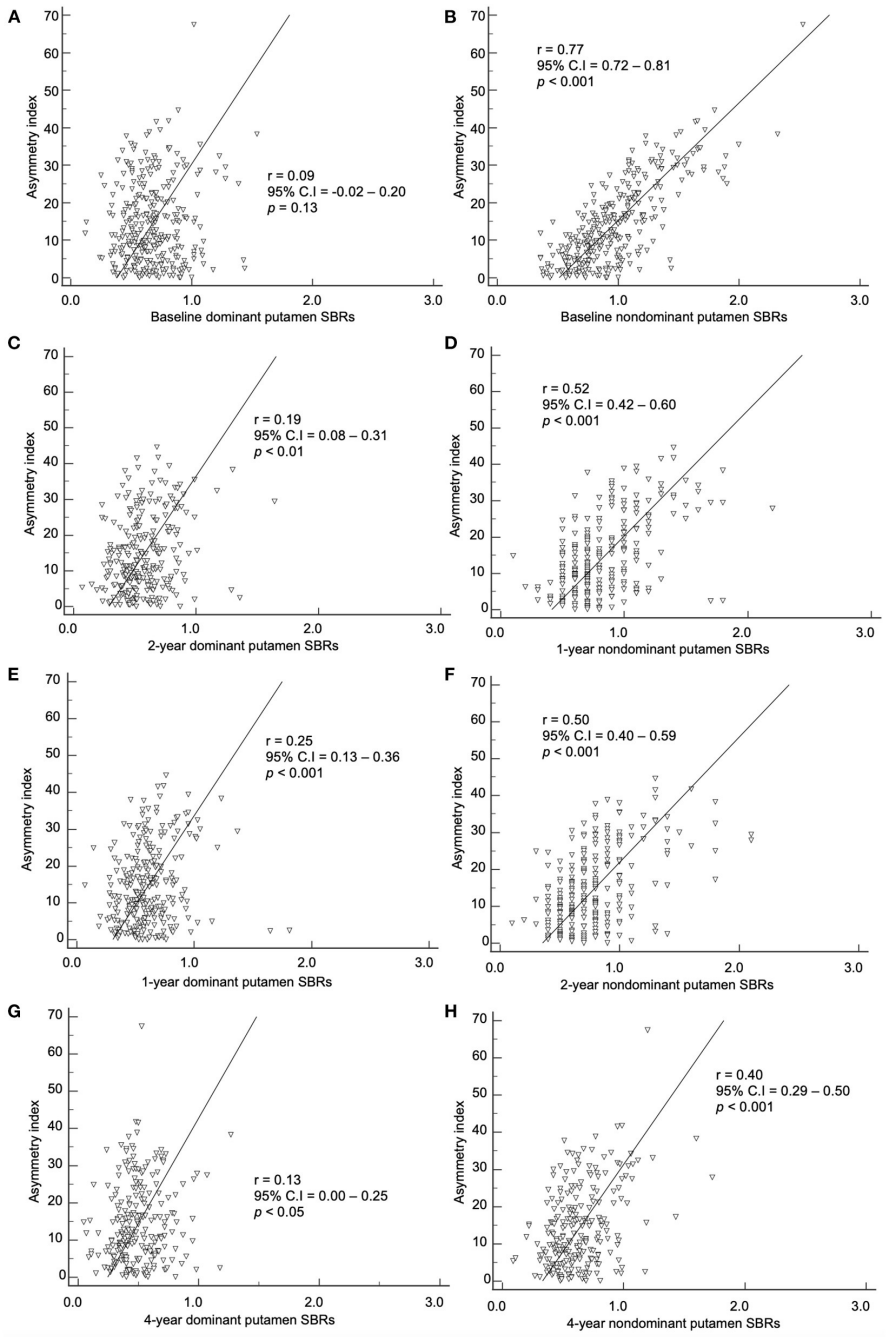
Correlation of the baseline asymmetry index of the putamen by method 2 with SBRs of **(A)** baseline, **(C)** 1-year, **(E)** 2-year, and **(G)** 4-year. Correlation of the baseline asymmetry index of the putamen by method 2 with SBRs of **(B)** baseline, **(D)** 1-year, **(F)** 2-year, and **(H)** 4-year.

The baseline asymmetry index by method 2 and baseline, 1-, 2-, and 4-year SBRs were correlated. The dominant hemisphere and non-dominant hemisphere were correlated separately (Figures 4, 5). The baseline asymmetry index *via* method 2 showed no significant correlation with the baseline, 1-, 2-, and 4-year SBRs of the dominant caudate. It showed a significant correlation with the baseline (*p* < 0.001), 1- (*p* < 0.001), 2- (*p* < 0.001), and 4-year (*p* < 0.001) SBRs of the non-dominant caudate. It showed a significant correlation with the 1- (*p* < 0.01), 2- (*p* < 0.001), and 4-year (*p* < 0.05) SBRs of the dominant putamen. It showed a significant correlation with the baseline (*p* < 0.001), 1- (*p* < 0.001), 2- (*p* < 0.001), and 4-year (*p* < 0.001) SBRs of the non-dominant putamen. For the non-dominant caudate and putamen SBRs, the correlation coefficient was largest among at baseline during the 4-year follow-up period.

### Correlation of baseline asymmetry index and symptoms

**Figure 6:**
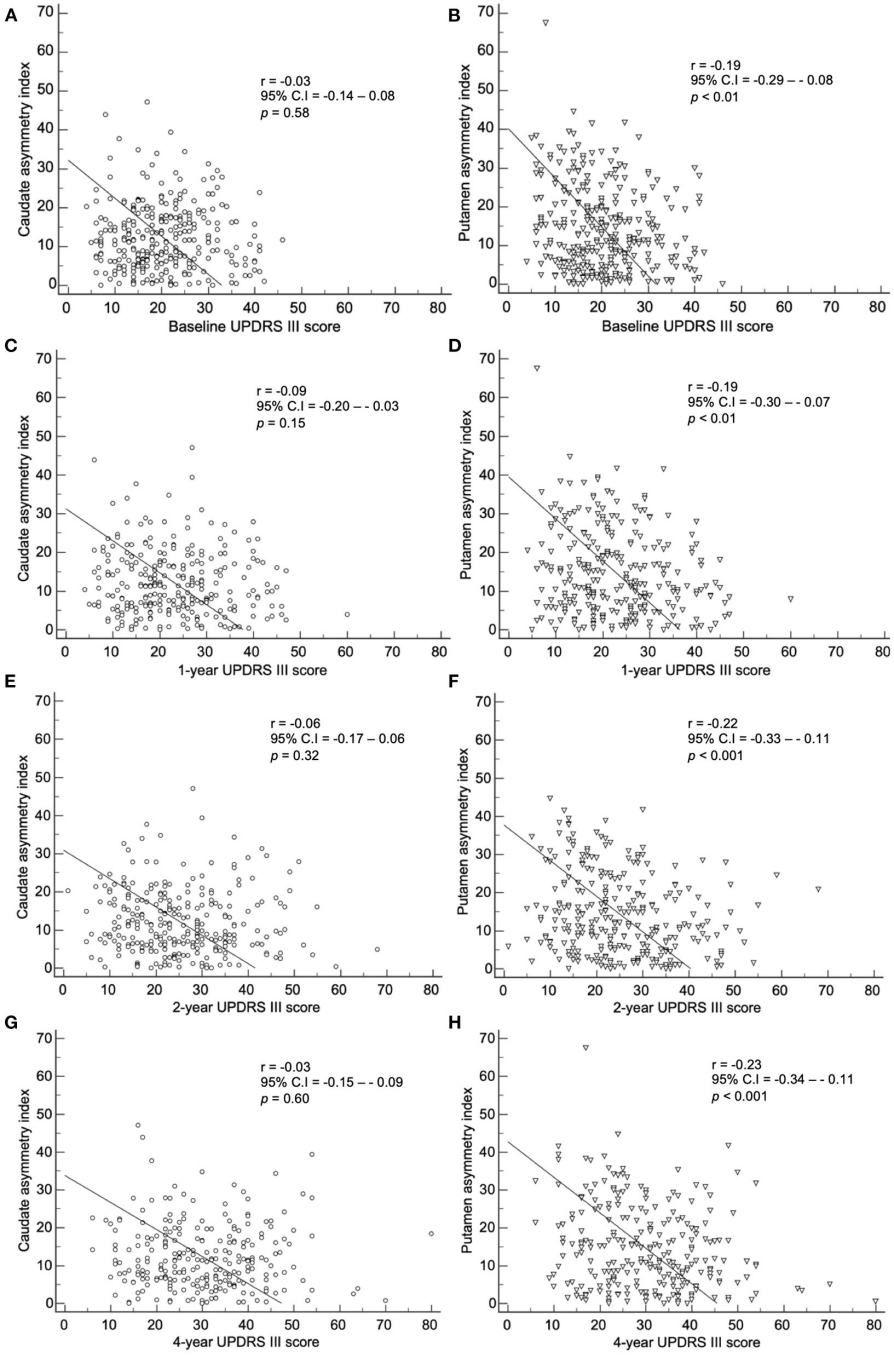
Correlation of the baseline asymmetry index of the caudate by method 2 with **(A)** baseline, **(C)** 1-year, **(E)** 2-year, and **(G)** 4-year UPDRS III scores. Correlation of the baseline asymmetry index of the putamen by method 2 with **(B)** baseline, **(D)** 1-year, **(F)** 2-year, and **(H)** 4-year UPDRS III scores.

The baseline asymmetry index by method 2 showed a correlation among the baseline, 1-, 2-, and 4-year UPDRS III scores (Figure 6). The baseline caudate asymmetry index by method 2 had no significant correlation with the baseline, 1-, 2-, and 4-year UPDRS III scores. The putamen asymmetry index showed a significant negative correlation with the baseline (*p* < 0.01), 1- (*p* < 0.01), 2- (*p* < 0.001), and 4-year (*p* < 0.001) UPDRS III scores, but the correlation coefficients were weak (< 0.30). The LEDD at 4-year follow-up period did not show a significant correlation with the 4-year UPDRS III scores.

**Figure 7:**
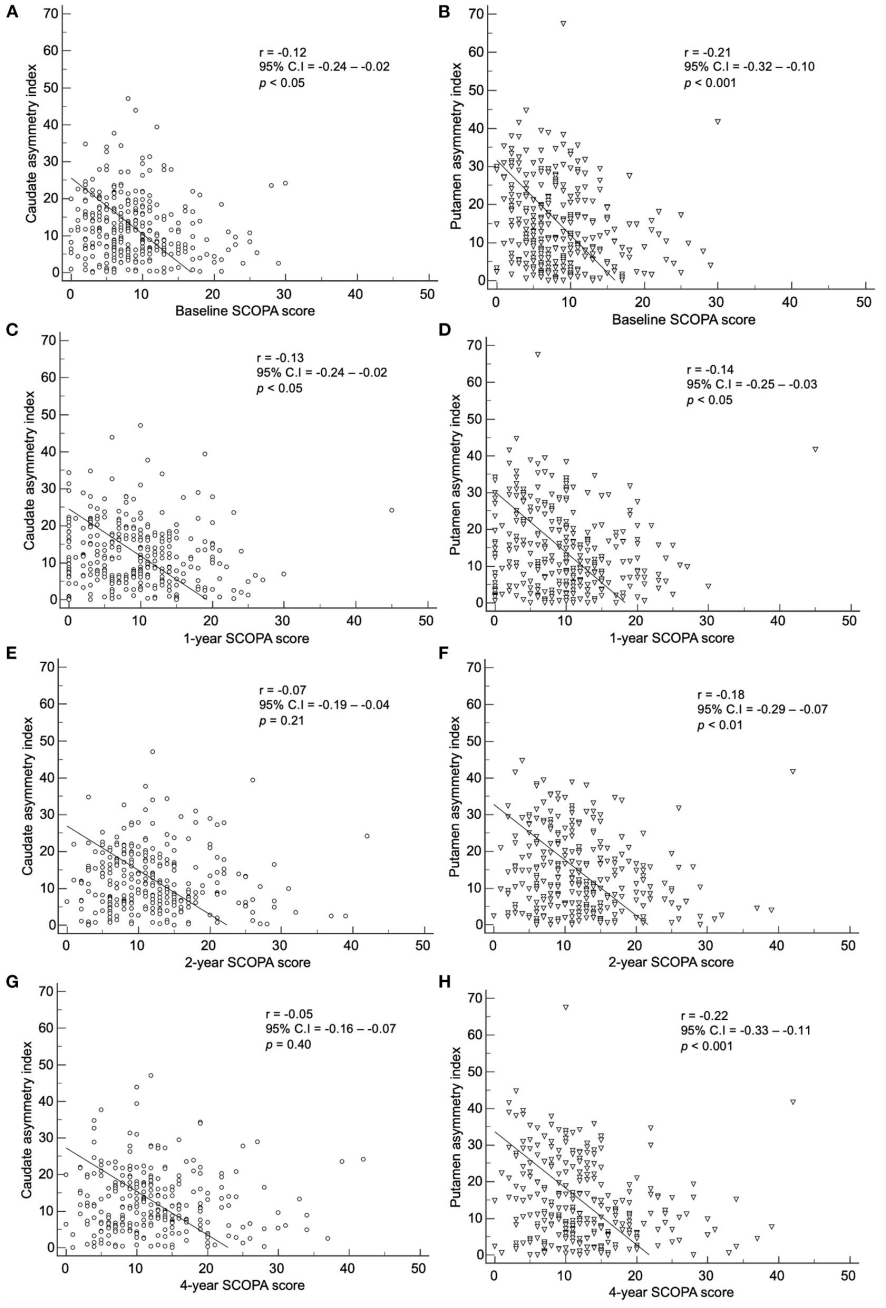
Correlation of the baseline asymmetry index of the caudate by method 2 with **(A)** baseline, **(C)** 1-year, **(E)** 2-year, and **(G)** 4-year SCOPA scores. Correlation of the baseline asymmetry index of the putamen by method 2 with **(B)** baseline, **(D)** 1-year, **(F)** 2-year, and **(H)** 4-year SCOPA scores.

By method 2, there was a correlation between the baseline index and baseline, 1-, 2-, and 4-year SCOPA scores (Figure 7). The baseline caudate asymmetry index *via* method 2 showed a significant negative correlation with the baseline (*p* < 0.05) and 1-year (*p* < 0.05) SCOPA scores. The putamen asymmetry index showed a significant negative correlation with the baseline (*p* < 0.001), 1- (*p* < 0.05), 2- (*p* < 0.01), and 4-year (*p* < 0.001) SCOPA scores; the correlation coefficients were weak (< 0.30).

**Figure 8:**
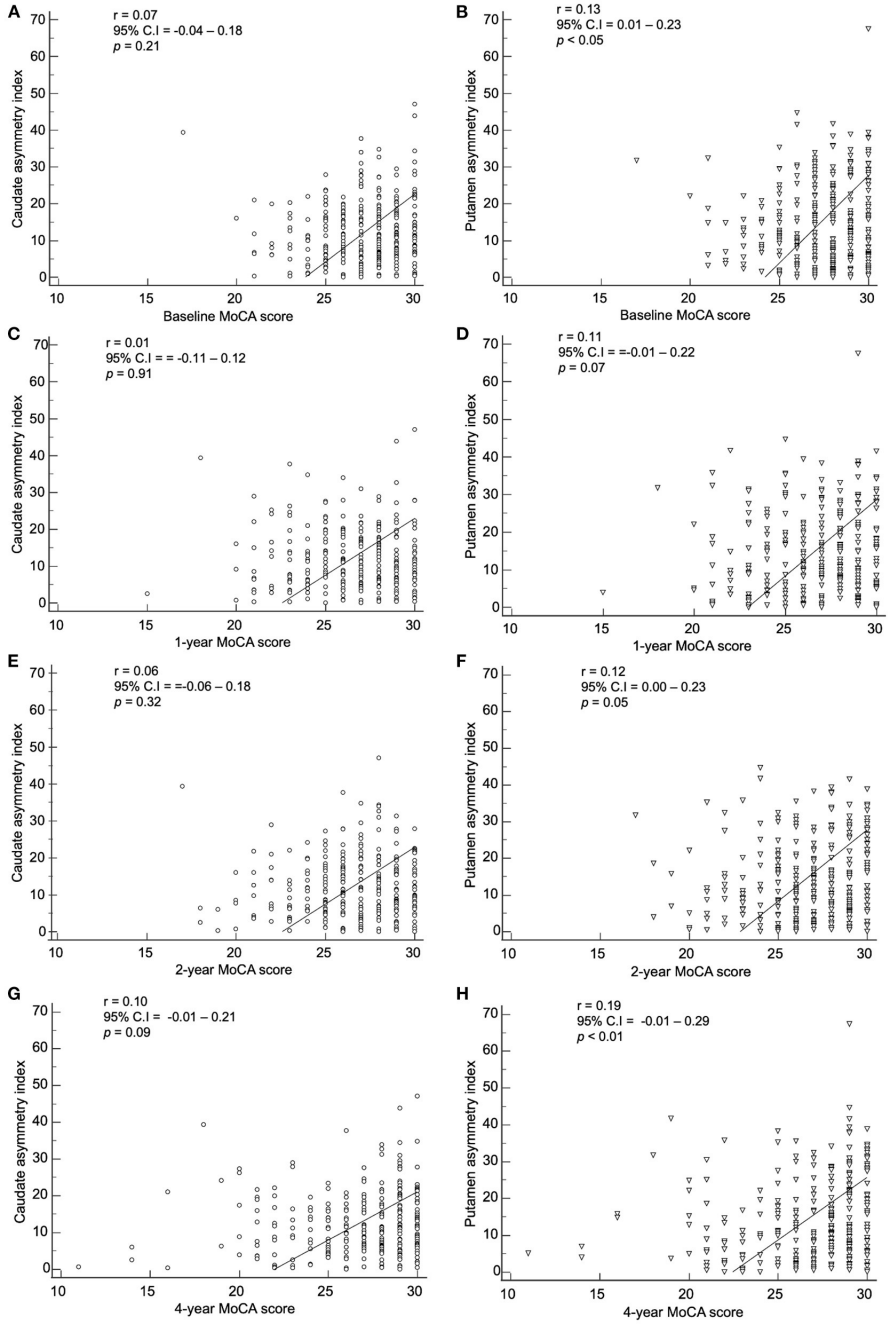
Correlation of the baseline asymmetry index of the caudate by method 2 with **(A)** baseline, **(C)** 1-year, **(E)** 2-year, and **(G)** 4-year MoCA scores. Correlation of the baseline asymmetry index of the putamen by method 2 with **(B)** baseline, **(D)** 1-year, **(F)** 2-year, and **(H)** 4-year MoCA scores.

The baseline index by method 2 showed a correlation with the baseline, 1-, 2-, and 4-year MoCA scores (Figure 8). The baseline caudate asymmetry index using method 2 had no significant correlation with the MoCA scores. The baseline putamen asymmetry index *via* method 2 showed a significant negative correlation with the baseline (*p* < 0.05) and 4-year (*p* < 0.01) MoCA scores, but the correlation coefficients were weak (< 0.20).

## Discussion

The present study evaluated serial changes in the asymmetry patterns of I-123 FP-CIT striatal binding during 4-year follow-up period. Previous studies that described the association between SBR asymmetry and clinical symptoms have only analyzed the baseline asymmetric index (5, 13, 15). This may be problematic, as each patient may be in different stages of the disease progression at the time of the study. Therefore, to use the SBR asymmetry index as a prognostic biomarker, we need to know the stage of disease progression for each patient at the time of study enrollment and evaluate the changes of SBR asymmetry index throughout the follow-up period. Therefore, in this article, we described the SBR asymmetry index for patients using two different methods during 4-year follow-up period.

The SBR asymmetry index that was used in previous studies (asymmetry index by method 1) was calculated as the absolute ratio of difference to average striatal SBRs (7, 11–16). Although there was a significant decrease in the putaminal asymmetry index using method 1 after the 1-year follow-up mark, there was no notable pattern of changes during the entire follow-up period. The dopaminergic denervation throughout the progression of PD is known to occur exponentially; therefore, a decrease in the SBR asymmetry index is higher in the earlier stages of the disease (17). This means that the absolute difference between right and left SBRs has a different clinical manifestation depending on the stage of PD progression. Method 1 does not reflect this difference of SBRs that is due to patient's stage of disease progression. However, method 2 in this study showed a persistent decrease in the asymmetry throughout the 4-year follow-up period. Using method 2, the putaminal asymmetry index decreased throughout the 4 years of follow-up. This indicates that the asymmetry index should be defined by considering the relative decrease of striatal SBRs compared to the age-matched normal baseline values. Also, the striatal SBRs in NC subjects are known to decrease with age (16, 18, 19). Therefore, we used the ratio to age-matched NC subjects' SBR values. We suggest to use this new asymmetry index for future studies investigating the asymmetric binding of I-123 FP-CIT.

In our study, the baseline asymmetry index by method 2 showed a correlation with non-dominant hemisphere SBRs from baseline to the 4-year follow-up mark. The correlation coefficient was stronger in the baseline period (0.77 for non-dominant putamen) than the 4-year follow-up period (0.40 for non-dominant putamen). As PD progress during the 4 years of follow-up, the asymmetry index decreases, while the non-dominant putamen SBR also decreases. During the progression of PD, the non-dominant hemisphere's SBR decreases more than the dominant hemisphere, leading to a decrease in asymmetry. The non-dominant hemisphere has a higher SBR compared to the dominant hemisphere. Therefore, higher asymmetry at baseline indicates a higher potential for dopaminergic denervation with further progression.

Despite several previous study evaluating the asymmetry pattern of motor symptoms, results remain inconclusive and inconsistent. A previous study analyzing motor symptoms according to age and symptom duration at the time of observation revealed no significant difference in asymmetry (20). Another prospective study analyzing motor asymmetry revealed that no specific motor symptoms converted to a symmetric pattern during the 5-year follow-up period (4). However, the PROfiling PARKinson's disease (PROPARK) study showed that most patients with symmetric motor symptoms at baseline remained stable throughout the 5-year follow-up period, while significant portion of patients with asymmetric motor symptoms at baseline later showed symmetric motor symptoms (5). The different suggestion among previous studies may be due to methodological heterogeneity, such as retrospective cross-sectional, between-subject analysis and prospective, within-subject analysis. In our study, the SBR asymmetry index significantly decreased within patients during the 4-year follow-up period. This gives weight to the conversion of asymmetric motor symptoms to symmetric motor symptoms during the progression of PD.

The correlation between baseline asymmetry index by method 2 and clinical symptoms were weak in our study. There was no statistical significance or the correlation coefficient was too low with UPDRS III scores, SCOPA scores, and MoCA scores. Fiorenzato et al. claimed that PD patients with right predominance exhibited motor dysfunction with greater severity, and patients with left predominance showed greater severity in cognitive impairment (12). However, in our study, when the asymmetry index was correlated with SBRs in both right and left hand predominant PD patients, neither group showed significant correlation (data not shown). Since the deterioration of dopaminergic innervation progresses exponentially, SBRs are usually already decreased significantly in the preclinical stage (21, 22). Therefore, the aggravation of dopaminergic denervation and the deterioration of motor, autonomic, and cognitive symptoms have different starting points. This may explain the weak correlation.

Lastly, we investigated the relationship between the asymmetry index with clinical subtypes and gender. Due to the heterogenous symptomatic manifestation of PD, investigators have given effort to identify homogenous subtypes and provide a better understanding of the pathophysiology. In our study, the asymmetry index of the TD subtype had the most significant decrease, compared to the indeterminant and PIGD subtypes. It can be referred that patients with dominant unilateral motor symptoms during symptomatic presentation tend to have a higher asymmetry index, which decreases during the progression of disease. However, it has been reported that significant portion of PD patients convert to the PIGD subtype from the TD subtype during progression (23). Therefore, it would need cautious interpretation when comparing asymmetry index among different clinical subtypes. In our study, there were significant differences of SBRs of the NC group according to gender for both caudate and putamen, which was not the case for the PD group. Here, we performed an age-matched comparison of the SBRs, and with no gender-matched comparison. There were no significant differences of asymmetry indices according to gender. While age-related decline of I-123 FP-CIT uptakes have been proven in multiple previous studies along with the age-related decline of dopamine transporter activity in post-mortem studies, there seems to be several controversies for the gender related difference of I-123 FP-CIT uptakes. Some studies have reported higher SBRs in females of NCs (24, 25), while others reported no difference according to gender (26, 27). Furthermore, this gender related difference of SBRs were suggested to be dependent on the image reconstruction method (11, 28). Further studies with an age- and gender-matched comparison with a larger number of NCs may be needed to clarify this issue.

There are several limitations to be aware of in the present study. First, the PPMI is a multicenter clinical trial, acquiring I-123 FP-CIT SPECT images from different gamma scanners. Although the PPMI data lab performs quality control, image quality variations among institutions cannot be avoided. Our study group performed a visual inspection of the I-123 FP-CIT SPECT images to exclude significant variations. Second, as aforementioned, we lost some patients throughout the 4-year follow-up period, resulting in 239 final patients, out of the initial 304 patients at the onset of the study. Also, a larger number of participants for the NC group may be advisable for an age-matched comparison. Third, while the PD cohort of the PPMI is consisted of sporadic PD patients (29), the genetic cohort is consisted of patients with confirmation of LRRK2, GBA or SNCA mutation. Since the genetic cohort does not provide sufficient I-123 FP-CIT follow-up data, future studies may be needed to investigate the effect of genetic mutation on I-123 FP-CIT binding asymmetry. Fourth, PPMI performs participant evaluation at certain time points from enrollment, not at certain time points according to symptom duration. Therefore, each PD patients have different symptom durations at the follow-up periods. Standard deviation for the symptom duration of PD patients was 1.8 years, which might affect the results of serial comparison. Lastly, while drug-naïve patients were enrolled, LEDD during the follow-up could have affected the correlation of UPDRS III scores and asymmetry indices.

In conclusion, we suggest a novel asymmetry index in association with age-matched normal SBR values. The asymmetry index decreases during the progression of PD, and have significant correlation with the SBRs of the non-dominant hemisphere. This novel asymmetry index may be adopted for future studies.

## Data availability statement

The original contributions presented in the study are included in the article/Supplementary material, further inquiries can be directed to the corresponding author/s.

## Ethics statement

The PPMI study was approved by the local Institutional Review Boards of respective institutions (a full list is available as a supplementary file or at the following link https://www.ppmi-info.org/about-ppmi/ppmi-clinical-sites). Written informed consent were obtained from each participant at enrollment, in accordance with the Declaration of Helsinki. All methods were performed in accordance with the relevant guidelines and regulations.

## Author contributions

EJ, MS, and YS designed the study and drafted the final manuscript. JL, S-KH, and SH contributed to statistical analysis. EJ and YS analyzed and interpreted the patient data. All authors contributed to the article and approved the submitted version.

## Funding

This research was supported by the National Research Foundation of Korea (Grants NRF-2019M3C7A1032718).

## Conflict of interest

The authors declare that the research was conducted in the absence of any commercial or financial relationships that could be construed as a potential conflict of interest.

## Publisher's note

All claims expressed in this article are solely those of the authors and do not necessarily represent those of their affiliated organizations, or those of the publisher, the editors and the reviewers. Any product that may be evaluated in this article, or claim that may be made by its manufacturer, is not guaranteed or endorsed by the publisher.
