# Supplementary material for: Serial changes of I-123 FP-CIT SPECT binding asymmetry in Parkinson's disease: Analysis of the PPMI data
Source: Front Neurol. 2022 Sep 1;13:976101. doi: 10.3389/fneur.2022.976101 (PMC9474999; doi:10.3389/fneur.2022.976101)
Supplement: Supplementary file 1 [file Data_Sheet_1.docx]

**Institutional Review Boards**

Ann Arbor, MI University of Michigan

Athens, GREECE National and Kapodistrian University of Athens

Atlanta, GA Emory University

Aurora, CO University of Colorado

Baltimore, MD Johns Hopkins University

Barcelona, SPAIN Hospital Clinic de Barcelona

Birmingham, AL University of Alabama at Birmingham

Boca Raton, FL PD and Movement Disorders Center of Boca Raton

Boston, MA Massachusetts General Hospital

Boston, MA Boston University

Chicago, IL Northwestern University

Cincinnati, OH University of Cincinnati

Cleveland, OH Cleveland Clinic Foundation

Gainesville, FL University of Florida

Houston, TX Baylor College of Medicine

Innsbruck, AUSTRIA Innsbruck University

Kansas City, KS University of Kansas Medical Center

Kassel, GERMANY Paracelsus-Elena Clinic Kassel/University of Marburg

Las Vegas, NV Lou Ruvo Center for Brain Health

London, UK Imperial College London

Los Angeles, CA Keck School of Medicine of the University of Southern California

Luebeck, GERMANY Lübeck University

Montreal, Canada Montreal Neurological Institute-Hospital

New Haven, CT Institute for Neurodegenerative Disorders

New York, NY Columbia University Medical Center

New York, NY Beth Israel Medical Center

New York, NY NYU Langone Health

Newcastle upon Tyne, UK Clinical Ageing Research Unit Newcastle

Nijmegan, NETHERLANDS Radboud University

Ottawa, Canada The Ottawa Hospital

Oxford, UK John Radcliffe Hospital Oxford and Oxford University

Paris, FRANCE Pitié-Salpêtrière Hospital

Philadelphia, PA University of Pennsylvania

Phoenix, AZ Barrow Neurological Institute

Pittsburgh, PA University of Pittsburgh

Portland, OR Oregon Health & Science University

Rochester, NY University of Rochester

Salerno, ITALY University of Salerno

San Diego, CA University of California at San Diego

San Francisco, CA University of California, San Francisco

San Sebastian, SPAIN Hospital Universitario Donostia

Scottsdale, AZ Mayo Clinic Arizona

Seattle, WA VA Puget Sound Health Care System

Sun City, AZ Arizona Parkinson’s Disease Consortium-Banner Sun Health Research Institute

Tampa, FL University of South Florida

Tel Aviv, ISRAEL Tel Aviv Sourasky Medical Center

Toronto, Canada Toronto Western Hospital

Trondheim, NORWAY St. Olavs Hospital

Tubingen, GERMANY Universitat Tubingen, DZNE und Hertie-Institut fur Klinische Hirnforschung
